# Pathologies cutanées vues au laboratoire d'anatomie pathologique à Lomé, Togo

**DOI:** 10.11604/pamj.2015.21.41.6219

**Published:** 2015-05-21

**Authors:** Tchin Darre, Abas Mouhari-Toure, Bayaki Saka, Efoé-ga Yawod Olivier Amouzou, Sassil Dare, Dadja Essoya Landoh, Koffi Amegbor, Palokinam Pitché, Gado Napo-Koura

**Affiliations:** 1 Laboratoire d'Anatomie et Cytologie Pathologiques, CHU Sylvanus Olympio, Lomé, Togo; 2 Service de Dermatologie, CHU Sylvanus Olympio, Lomé, Togo; 3 Services de Chirurgie, CHU Sylvanus, Lomé, Togo; 4 Division de l'Epidémiologie, Ministère de la Santé, Lomé, Togo

**Keywords:** Pathologie cutanée, histopathologie, Togo, skin pathology, histopathology, Togo

## Abstract

**Introduction:**

Les affections cutanées restent encore un problème de santé publique dans la majorité des pays en développement. Notre étude s'est fixée comme objectif de déterminer les aspects épidémiologiques et histologiques des dermatoses au Togo.

**Méthodes:**

Il s'agissait d'une étude descriptive et transversale portant sur les cas de dermatoses diagnostiquées de 2002 à 2013 (10 ans) au laboratoire d'anatomie pathologique (LAP) du CHU- Sylvanus Olympio. Tous les cas d'examen portant sur un prélèvement de peau (biopsie, exérèse, pièces opératoires) ont été colligés de 2002 à 2013 à partir des données des registres dudit laboratoire.

**Résultats:**

Au cours de la période d’étude, 1119 (7,6%) des 14720 prélèvements reçus au LAP étaient des prélèvements de peau, ce qui correspond à une fréquence annuelle de 111,9 prélèvements. L’âge moyen des patients dont les prélèvements de peau appartenaient était de 35,4 ans et le sex-ratio (H/F) de 1,39. Au plan histologique, les lésions cutanées étaient reparties en dermatoses non tumorales (390 cas, 34,8%), dermatoses tumorales et pseudo tumorales (607 cas, 54,2%) et des dermatoses de diagnostic incertain (122 cas, 10,9%). La lèpre (95 cas, 8,5%) et le carcinome épidermoïde (134 cas, 11,9%) étaient les types histologiques les plus fréquents.

**Conclusion:**

Les dermatoses tumorales et pseudo tumorales font plus l'objet de demande d'examen anatomopathologique au Togo, ce qui s'explique par la hantise de tumeurs malignes devant toute dermatose tumorale. L'amélioration du plateau technique du LAP (immunohistochimie, immunofluorescence directe) permettra d'accroitre ses capacités diagnostiques.

## Introduction

Les affections cutanées restent un problème de santé publique dans la majorité des pays en développement. En effet, ces affections constituent un motif fréquent de consultation en Afrique Noire et figurent parmi les 5 causes de morbidité et de perte de capacité de travail dans cette région [1, 2]. Malgré l'importance des maladies cutanées en région tropicale, la dermatologie est toujours un parent pauvre de la médecine moderne en Afrique Noire à cause du manque de structures et surtout des médecins spécialistes [3, 4]. La dermatologie africaine est encore dominée par la pathologie infectieuse, mais depuis une quinzaine d'années on observe l’émergence surtout en zone urbaine des dermatoses telles que les eczémas et les dyschromies jadis qualifiées de «pathologies des pays du Nord» et des pathologies cutanées liées à l'infection à VIH/Sida [3, 5, 6]. En Afrique Noire, il existe des particularités cliniques et épidémiologiques des affections cutanées observées, et les dermatologues ont souvent recours à l'anatomopathologie, qui apporte des éléments de diagnostic sûr dans de nombreux cas. Cependant, la dermatopathologie est embryonnaire dans ces pays en développement du fait surtout du plateau technique limité dans la plupart de ces pays. L'objectif de notre travail était de décrire les aspects épidémiologiques et histologiques des dermatoses diagnostiquées au laboratoire anatomopathologique (LAP) du CHU Sylavanus Olympio.

## Méthodes

Il s'agissait d'une étude transversale et descriptive sur tous les dossiers (registres et comptes rendus d'examens) portant sur les pathologies cutanées histologiquement diagnostiquées au LAP du CHU- Sylvanus Olympio de Lomé de janvier 2002 à décembre 2013 soit une période de 10 ans. Au cours de cette période, tous les cas d'examen portant sur un prélèvement de peau (biopsie, exérèse, pièces opératoires) ont été colligés à partir des données des registres dudit laboratoire. Les résultats et comptes rendus d'examen de tous les cas colligés à partir des registres avaient fait l'objet d'une collecte à l'aide d'une fiche préétablie. Les techniques d'examen des prélèvements étaient essentiellement constituées de coupes incluses en paraffine (56°-60° C), puis colorées à l'hématéine éosine ou par des colorations spéciales (PAS, GOMORY-GROCOTT, ZIELH). Les variables étudiées étaient les données épidémiologiques (sexe, âge, provenance) et histologique (type histologique). Le traitement et l'analyse des données ont été réalisés à l'aide du logiciel Epi Info version 3.5.1.

### Considération éthique

Cette étude a reçu l'autorisation du Chef du département des laboratoires pour être conduite. Vu qu'il s'agissait de dépouillement des dossiers, le consentement des patients n’était pas requis. Toutefois lors du dépouillement et la collecte de données les noms des patients n'avaient pas été collectés afin de préserver la confidentialité.

## Résultats

### Aspects épidémiologiques

Au cours de la période d’étude, 1119 (7,6%) des 14720 prélèvements reçus au LAP étaient des prélèvements de peau. La fréquence annuelle des dermatoses était de 111,9 cas. L’âge des patients dont les prélèvements sont étudiés variait entre 3 et 99 ans avec un âge moyen de 35,4 ans; le sex-ratio (H/F) était de 1,4. Les prélèvements provenaient des formations sanitaires de la capitale dans 932 cas (83,6%), des formations sanitaires de l′intérieur du pays dans 8,9% et des formations sanitaires non précisées dans 7,5% des cas. Les prélèvements étaient constitués de 1100 biopsies cutanées et de 19 pièces opératoires.

### Aspects anatomopathologiques

Les résultats d'examens étaient repartis en dermatoses non tumorales (390 cas, 34,9%), dermatoses tumorales et pseudo tumorales (607 cas, 54,2%) et des dermatoses de diagnostic incertain (122 cas, 10,9%). Les dermatoses non tumorales étaient constituées de dermatoses infectieuses (150 cas, 38,5%) dominées par la lèpre dans 63,3% des cas et des dermatoses non infectieuses (240 cas, 61,5%) dont l'eczéma (30%) était le type histologique majoritaire (Tableau 1 et Tableau 2). L’âge moyen des patients qui ont présenté des dermatoses non tumorales était 24,8 ans avec un sex-ratio H/F de 0,9. Les tumeurs cutanées étaient classées en tumeurs bénignes et pseudotumeurs dans 330 cas et en tumeurs malignes dans 150 cas. Les tumeurs bénignes survenaient à un âge moyen de 32,3 ans et étaient de nature épithéliale dans 148 cas, mésenchymateuse dans 173 cas et pigmentaires dans 9 cas. Parmi ces tumeurs bénignes, les types histologiques les plus fréquents étaient le botriomycome (73 cas) et les verrues (62 cas) (Tableau 3). Les tumeurs malignes étaient observées à un âge moyen de 44,8 ans, avec une prédominance masculine (162 cas, 67,5%). Les groupes histologiques étaient les carcinomes (140 cas, 54,5%), les sarcomes (98 cas, 35,4%), le mélanome (29 cas, 10,4%), le lymphome (4 cas, 1,5%) et les tumeurs cutanées secondaires (6 cas, 2,2%). Les carcinomes étaient dominés par le carcinome épidermoïde (134 cas, 95,7%), et les sarcomes par la maladie de Kaposi (52 cas, 53,1%) (Tableau 4). L’âge moyen de survenue du carcinome épidermoïde était de 49,5 ans, localisé de façon préférentielle aux membres inférieurs (73 cas, 54,5%) et à la face (23 cas, 17,2%). La maladie de Kaposi survenait à un âge moyen de 43 ans, plus chez le e sujet de sexe masculin (dans 47 cas), localisée dans 80% des cas aux membres inférieurs. Le mélanome était majoritairement observé chez les patients de sexe féminin (18 cas, 62%) et essentiellement localisé aux plantes des pieds (29 cas, 100%). Tous les cas de lymphomes cutanés observés étaient d'origine T. Les tumeurs cutanées malignes secondaires étaient localisées au tronc (5 cas) et au mamelon (1 cas). La tumeur primitive était l'adénocarcinome dans 4 cas, carcinome épidermoïde dans un cas et un cas de maladie de Paget de mamelon.

**Tableau 1:** Répartition des dermatoses infectieuses

Affection observée	Nombre de cas	Pourcentage
**Affections bactériennes et mycobactériennes (n= 120)**		
Lèpre	95	63,4
Tuberculose cutanée	11	7,3
Pyodermite	10	6,7
Impétigo	2	1,3
Pian	2	1,3
**Affections parasitaires (n= 18)**		
Onchocercose	15	10
Cysticercose	3	2
**Affections mycosiques (n= 8)**		
Mycétome	6	4
Histoplasmose africaine	2	1,3
**Infection Virale (n= 4)**		
Condylome vénérien	4	2,7
Total	**150**	**100%**

**Tableau 2:** Répartition des dermatoses non tumorales et non infectieuses

Variété histologique	Nombre de cas	Pourcentage (%)
**Dermatoses à prédominance épidermique (n=133)**		
Eczéma	72	30
Psoriasis	35	13,3
**Pemphigus et pemphigoïde bulleuse**	11	4,6
Ichtyoses	6	2,5
Vitiligo	6	2,5
Eczématides	2	0,8
Epidermolyse bulleuse	2	0,8
Incontinentia Pigmenti	2	0,8
**Dermatoses à prédominance dermique (n=47)**		
Granulome inflammatoire	18	7,6
Sclérodermie	14	5,8
Granulome annulaire	5	2,1
Sarcoïdose	4	1,7
Dermatomyosite	4	1,7
Dermatite de surcharge (Tophus goutteux)	2	0,8
**Dermatoses à prédominance dermo-hypodermique (N= 60)**		
Lichen Plan	35	14,6
Lupus érythémateux chronique	8	3,3
Toxidermie	5	2,5
Kératodermie	4	1,7
Lichen scléro- atrophique	4	1,7
Erythème polymorphe	1	0,4
Parapsoriasis en plaque	1	0,4
Prurigo nodulaire de Hyde	1	0,4
Dermatite herpétiforme	1	0,4
Total	**240**	**100%**

**Tableau 3:** Répartition des tumeurs et pseudotumeurs bénignes cutanées

Variété histologique	Nombre de cas	Pourcentage
**Tumeurs bénignes épithéliales (n=148)**		
Verrues	62	18,7
Kyste épidermique	30	9
Molluscum contagiosum	27	8,2
Papillome corné	21	6,3
Tricho-épithéliome solitaire	3	0,9
Adénome sébacé	2	0,6
Syringome	2	0,6
Kérato-acanthome	1	0,3
**Tumeurs bénignes mésenchymateuses (n=173)**		
Botriomycome	73	22,1
Cheloïde	44	13,3
Lipome	22	6,7
Angiome	12	3,6
Lipofibrome	9	2,7
Fibrome	6	1,8
Hémangiome	4	1,6
Schwanome	2	0,6
Dermatomyome	1	0,3
**Tumeur pigmentaire (n= 9)**		
Naevus	9	2,7
Total	**240**	**100%**

**Tableau 4:** Répartition cancers cutanés selon le groupe et le type histologique

Variété histologique	Nombre de cas	Pourcentage (%)
**Carcinomes (N=140)**		
Carcinome épidermoïde	134	50,2
Carcinome basocellulaire	5	1,8
Carcinome métatypique	1	0,3
**Sarcomes (N=98)**		
Maladie de Kaposi	52	19,4
Fibrosarcome	35	13,1
Angiosarcome	5	1,8
Dermatofibrosarcome	4	1,4
Lymphangiosarcome	2	0,6
Mélanome	29	10,8
Lymphome T	4	1,4
Tumeurs cutanées secondaires	6	2,2
Total	**267**	**100%**

## Discussion

Notre laboratoire est le seul LAP publique du pays et reçoit les prélèvements provenant de tout le pays. Les demandes d'examens anatomopathologiques proviennent essentiellement de la capitale (83,6%), ce qui est conforme à la répartition et à l'accessibilité aux services de soins de santé. Notre étude semble être la première du genre au Togo, et bien que les dermatoses diagnostiquées dans le LAP ne reflètent pas l'ensemble des pathologies cutanées au Togo, elle permet de faire le point sur l'importance du LAP du CHU SO dans la prise en charge de ces dermatoses. Les pathologies cutanées observées dans le LAP sont très diverses, mais dominées par le groupe des affections tumorales et pseudotumorales. Les pathologies cutanées les plus rencontrées dans notre étude étaient le carcinome épidermoïde, la lèpre, le botriomycome et les eczémas.

### Données épidémiologiques

Dans notre étude, le CHU- Sylvanus Olympio de Lomé a adressé le plus grand nombre de pièces, montrant l′importance de ce centre dans notre pays. En effet, le CHU- Sylvanus Olympio est l′hôpital de référence au Togo, et le site d′implantation du LAP. Nous avons observé une fréquence élevée de la pathologie cutanée chez les sujets âgés de moins de 50 ans; ce résultat n′est qu′un reflet du profil démographique de la population de notre pays [7]. Il faut signaler par ailleurs que les dermatoses sont observées à tous les âges et dans les deux sexes. Nos résultats montrent une légère prédominance féminine, comparables à ceux de certains auteurs africains, notamment Pitché et al. au Togo, et Gimbel et Legesse en Ethiopie [1, 8].

### Données anatomopathologiques

Les dermatoses infectieuses représentaient seulement 13,4% de notre échantillon. Cette fréquence est largement plus basse que dans la population générale. En effet, des études antérieures sur les pathologies cutanées rencontrées dans les services de dermatologie et dans la population générale suggèrent que ces pathologies infectieuses sont largement plus fréquentes et sont au premier rang en Afrique subsaharienne [1, 5, 9]. En plus, les prélèvements de ces dermatoses infectieuses sont plus adressées au laboratoire de bactériologie et de mycologie. De plus, la plupart de ces pathologies infectieuses font plus l′objet, d′un diagnostic clinique; ce qui explique la fréquence basse dans notre étude. La lèpre constituait la dermatose d′origine bactérienne la plus fréquente et représentait 8,1% de notre échantillon; Pitche et al. avaient retrouvé une fréquence de 0,82% dans les motifs de consultation en dermatologie [1]. La place prépondérante de la lèpre qui est une Mycobactériose cutanée n′est pas étonnante dans notre étude par rapport à celle de Pitche et al. car la confirmation diagnostique de cette affection se base essentiellement sur l'histopathologie [10]. Bien que la prévalence de la lèpre soit en baisse à Lomé depuis 2 décennies, cette affection reste d'actualité en Afrique subsaharienne à cause de la persistance de rare cas de transmission, le changement de profil en faveur de la forme multibacillaire, et la fréquence élevée des rechutes [11, 12]. Les eczémas, le lichen plan et le psoriasis prédominent parmi les dermatoses non tumorales et non infectieuses rencontrées au LAP, ceci probablement à cause de leurs fréquences élevées en consultation dermatologique, de leurs nombreuses formes cliniques, et de leurs innombrables diagnostics différentiels rendant difficile le diagnostic clinique [1, 9]. Nous avons observé plusieurs variétés histologiques de tumeurs mésenchymateuses bénignes majoritairement dominées par le botriomycome et les chéloïdes. Nos résultats sont superposables à ceux de Pitché et al. qui avaient retrouvé 40 cas sur une période de 5 ans [1]. Nous avons retrouvé 44 cas de chéloïdes; Pitche et al. ont retrouvé dans une étude antérieure 234 cas [1]. C′est tumeurs sont plus diagnostiquées cliniquement; seuls les cas suspects de malignité font l′objet d′une biopsie pour l′examen anatomopathologique, plus souvent des cas d'exérèse complète, ce qui explique la faible fréquence de ces tumeurs retrouvées dans notre étude.

Les tumeurs bénignes épithéliales représentaient 24,4% de l′ensemble des tumeurs cutanées et 44,84% des tumeurs bénignes dominées par les verrues. L′âge moyen des patients présentant ces tumeurs bénignes retrouvées dans notre série est inférieur à celui qui est classiquement décrit dans la littérature où ces tumeurs surviennent autour de la cinquantaine [8]. Néanmoins, certains cas dits juvéniles ont été signalés par ces mêmes auteurs. Le molluscum contagiosum occupait le second rang après les verrues. Il s'agit d'une tumeur virale observée actuellement de manière préférentielle au cours du SIDA [8]. Les cancers cutanés ont longtemps eu la réputation d′être rares chez les sujets de peau noire. Cependant tous les types histologiques de cancers se retrouvent en Afrique Noire, mais à des fréquences différentes [13, 14]. Les carcinomes cutanés avec un taux de 50,5% de l′ensemble des tumeurs malignes cutanées, dominés par le carcinome spinocellulaire, représentent les tumeurs malignes les plus fréquentes rencontrées au Togo. Dans les séries européennes, le carcinome basocellulaire est le type histologique le plus rencontré [15]. La fréquence de carcinome basocellulaire est faible chez le noir africain: 2 à 16% contre 48 à 75% des cas chez les européens [16]. La prédominance de la maladie de Kaposi au sein des sarcomes, avec comme site cutané des membres a été retrouvé dans presque toutes les études [17]. L’épidémie du VIH a considérablement modifié l’épidémiologie de la maladie de Kaposi en Afrique subsaharienne avec une augmentation des formes liée au VIH-Sida [17, 18]. Le Dermatofibrosarcome de Darrier et Ferrand avait occupé la deuxième place des sarcomes comme l'avait déjà noté Saka et al. dans leur étude [14]. La rareté du mélanome dans notre étude a été rapportée dans plusieurs séries africaines, confirmant la faible fréquence de ce cancer chez les sujets à peau pigmentée [9, 19]. En plus, la particularité du mélanome chez l'Africain noire a été retrouvée dans notre étude: localisation préférentiellement aux plantes des pieds [19, 20]. Le nombre de cas assez faible, mais non exhaustif des métastases cutanées de notre étude, nous permet de conclure que la fréquence d'apparition des métastases est relativement rare dans notre pays, concordant avec les données de la littérature [12, 20]. Le nombre élevé de diagnostic incertain pourrait s'expliquer par une mauvaise technique du geste biopsique et surtout par l’étroitesse du plateau technique de notre laboratoire, souvent commun aux pays en développement [1, 5, 9].

## Conclusion

Notre étude concernant les dermatoses cutanées observées en 10 ans au LAP au Togo nous a permis de noter que les dermatoses tumorales et pseudo tumorales font plus l'objet de demande d'examen anatomopathologique au Togo. Les pathologies cutanées les plus fréquemment rencontrées sont le carcinome épidermoïde, la lèpre, le botriomycome, et les eczémas. Le carcinome épidermoïde et la maladie de Kaposi prédominent sur le tableau des cancers cutanés. L'amélioration du plateau technique du LAP (immunohistochimie, immunofluorescence directe) permettra d'accroitre les demandes d'examen notamment pour les dermatoses non tumorales.

## References

[1] Pitche P, Tchamdja S, Amanga Y, Tchangaï-Walla K (1997). Pathologie dermatologique en consultation hospitalière à Lomé (Togo). Nouv Dermatol..

[2] Caumes E (1998). Atlas De Dermatologie Tropicale.

[3] Caumes E (2003). The changing face of tropical dermatology. Bull Soc Pathol Exot..

[4] Morand JJ (2009). Dermatology in tropical zones: what future?. Med Trop (Mars)..

[5] Mahe A, Cisse I, Faye O, N'Diaye HT, Niamba P (1998). Skin diseases in Bamako (Mali). Int J Dermatol..

[6] Amerson EH, Maurer TA (2010). Dermatologic manifestations of HIV in Africa. Top HIV Med..

[7] Unité de recherche démographique (URD) (2004). Analyse de la situation en santé de la reproduction au Togo: Résultats de l'enquête dans les formations sanitaires.

[8] Gimbel DC, Legesse TB (2013). Dermatopathology practice in ethiopia. Arch Pathol Lab Med..

[9] Bissek AC, Tabah EN, Kouotou E, Sini V, Yepnjio FN, Nditanchou R (2012). The spectrum of skin diseases in a rural setting in Cameroon (sub-Saharan Africa). BMC Dermatol..

[10] Pinquier L (2011). Histopathology of leprosy. Ann Dermatol Venereol..

[11] Ekeke N, Chukwu J, Nwafor C, Ogbudebe C, Oshi D, Meka A (2014). Children and leprosy in southern Nigeria: burden, challenges and prospects. Lepr Rev..

[12] Saka B, Kombate K, Mouhari-Toure A, Amegan-Aho KH, Tchangai-Walla K, Pitche P (2008). Leprosy in Lome, Togo: retrospective study of 383 cases. Med Trop (Mars)..

[13] Nthumba PM, Cavadas PC, Landin L (2011). Primary cutaneous malignancies in sub-Saharan Africa. Ann Plast Surg..

[14] Saka B, Souley Z, Kombate K, Mouhari-Toure A, Akakpo S, Napo-Koura G (2010). Skin cancers in Togo: a 223-case series. Med Trop (Mars)..

[15] Leiter U, Eigentler T, Garbe C (2014). Epidemiology of skin cancer. Adv Exp Med Biol..

[16] Flohil SC, van der Leest RJ, Arends LR, de Vries E, Nijsten T (2013). Risk of subsequent cutaneous malignancy in patients with prior keratinocyte carcinoma: a systematic review and meta-analysis. Eur J Cancer..

[17] Gantt S, Kakuru A, Wald A, Walusansa V, Corey L, Casper C (2010). Clinical presentation and outcome of epidemic Kaposi sarcoma in Ugandan children. Pediatr Blood Cancer..

[18] Pitche PT, Kombate K, Owono F, Tchangai-Walla K (2007). Kaposi's sarcoma in a hospital setting in Lome (Togo): a study of 93 cases. Int J Dermatol..

[19] Pitche P, Napo-Koura G, Tchangai-Walla K (2005). Epidemiology of melanoma in Togo. Int J Dermatol..

[20] Pitche P, Napo K, Kpodzro K, Tchangai-Walla K (1996). Melanoma in Togo. A retrospective study over 20 years. Ann Dermatol Venereol..

